# Molecular Signatures of the Developing Hair Follicle

**DOI:** 10.1371/journal.pbio.0030366

**Published:** 2005-09-20

When a human baby enters the world, some 5 million hair follicles cover its body. For many, these follicles cease production with age, a fate that befalls both men and women, accounting for a multibillion-dollar hair growth industry, despite the products' limited success. Though the stages of hair development are understood, the molecular signals that guide the process are less clear.

Hair follicles develop from cells in the ectoderm, an embryonic epithelial layer that gives rise to the surface epidermis, and the underlying mesenchyme, connective tissue cells derived from the embryonic mesoderm. During fetal development, the spatial position and individual characteristics of the follicles (long versus short, for example) are determined. Once established, normal hair growth continues in cycles, with each follicle passing through three distinct stages before the hair is shed and the cycle begins anew. Both embryonic follicle formation and adult hair growth begin when mesenchymal dermal papilla (DP) cells, clustered beneath a single layer of epidermal cells, send the message to epithelial stem cells to make a hair follicle. Epithelial stem cells then send their progeny, epithelial matrix cells, to surround the DP cells and trigger the sequence of events that culminates in new hair.

To shed light on the molecular program that drives these mesenchymal–epithelial interactions, Michael Rendl, Lisa Lewis, and Elaine Fuchs used a cell-sorting technique that allowed them to isolate pure populations of DP cells along with populations of four neighboring cell types. Using microarrays to analyze the gene expression profiles of the different cell populations, the authors identified molecular signatures for the DP and its niche, including a group of little-studied genes linked to hair disorders. With these molecular signatures, researchers can begin to analyze each gene's role in hair development and growth.

To get the purified cell populations for their studies, the researchers used an innovative combination of existing techniques, and took advantage of the underlying biology of the hair follicle. In mice, hair begins to grow on the animal's back around 15 days in utero, and is fully formed roughly four days after birth. After the matrix cells envelop the DP, they proliferate, migrate upward, and differentiate into the hair shaft and the inner root sheath (IRS), which surrounds the hair. The IRS is covered by an outer root shaft (ORS), and the whole structure is enclosed by a membrane that separates the skin epithelium from the dermis and the DP. (Melanocytes, which determine hair color, sit just above this membrane.)

To extract cells from the DP along with its neighboring cells, Rendl et al. used transgenic mice and a cell-sorting technique called fluorescent-activated cell sorting, so they could retrieve the different cell types from the backs of the mice. Mice expressing red fluorescent protein (RFP) were bred with mice expressing green fluorescent protein (GFP). Because both reporters express the fluorescent proteins under known conditions—the promoter used to control RFP is typically expressed in DP cells, and the promoter used to control GFP is expressed in matrix and ORS cells—they serve as an initial filter in cell sorting.

Follicles were isolated from the backs of four-day-old mice, and five populations of cells—matrix, ORS, melanocytes, DP, or dermal fibroblasts (connective tissue cells)—were sorted based on whether or not they expressed GFP or RFP and whether they expressed known cell-surface markers. The authors were most interested in identifying the unique features of the cells that initiate the hair development program, the DP cells. They were sure the cells were from the DP because they expressed high levels of four known DP markers and triggered hair growth on mutant *Nude* mice, which only DP cells can do (along with ubiquitous keratinocytes).

Microarray analysis of the gene activity of the different cell types found that 4,000 genes were common to all the cell types, forming the “molecular backbone” of basic cellular functions. Just 150–300 genes showed elevated expression in any one cell type, forming the molecular signature for each population. Because the authors' DP signature genes differed substantially from those found in previous studies, they went on to verify protein expression in the cells, confirming that the signature accurately reflected the DP profile. Many of the signature genes are involved in genetic hair disorders, confirming the functional significance of the signatures, while others encode novel proteins involved in transcription, cell signaling, and cell adhesion. This subset provides a window into the special roles carried out by the different cells at the genomic level, and will help future studies explore their function in hair biology. [Fig pbio-0030366-g001]

**Figure pbio-0030366-g001:**
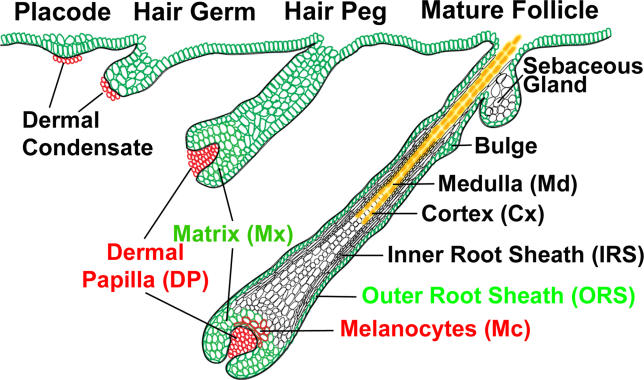
Having identified the molecular signatures of the dermal papilla and its neighboring cells, researchers can now study how these genes influence hair development and growth

Developmental biologists have made great progress in understanding the molecular agents of hair morphogenesis since Margaret Hardy called the hair follicle a “treasure waiting to be discovered” just over a decade ago. By identifying unique molecular profiles of the DP and its cohorts, this study has paved the way for revealing all the hair follicle's secrets, one signal at a time. For more on skin and hair follicle development, see the accompanying Primer by Sarah Millar (DOI: 10.1371/journal.pbio.0030372). *—Liza Gross*

